# Cytotoxic Lesion of the Corpus Callosum Caused by Puumala Hantavirus Infection

**DOI:** 10.5334/jbsr.1616

**Published:** 2019-01-21

**Authors:** Olivier Lebecque, Nicolas Mulquin, Michaël Dupont

**Affiliations:** 1 Université catholique de Louvain, CHU UCL Namur, Department of Radiology, 1 Avenue Dr G Thérasse, 5530, Yvoir, BE

**Keywords:** hantavirus, puumala, clocc, corpus, callosum, splenial, reversible, cytotoxic, edema

## Abstract

We report the case of a 45-year-old male referred to our hospital with fever, asthenia, visual disturbances and increasing headaches. Diffusion-weighted imaging of the brain showed high signal intensity in the splenium of corpus callosum with low apparent diffusion coefficient values. Diagnosis of cytotoxic lesion of corpus callosum was made with Puumala Hantavirus infection serologically confirmed and should not be mistaken for ischemia. Patient was discharged 8 days after admission and imaging findings had resolved 3 weeks later.

## Introduction

Puumala hantavirus (PUUV) along with its host, the bank vole, is found all over Europe, excluding the Mediterranean coastal regions and most of the Iberian Peninsula and Greece. Infection with hantavirus occurs predominantly after inhalation of contaminated aerosolized excreta of infected bank voles. Risk factors include living close to forested areas and exposure to rodents. In Europe, PUUV is the most common cause of hemorrhagic fever with renal syndrome. It is known to cause a generally mild disease, nephropathia epidemica, starting abruptly with initially influenza-like symptoms such as fever and headache. Acute renal failure with anuria or oliguria usually begins on days 3–4 and may require dialysis treatment [1,2]. Thrombocytopenia is common but bleeding symptoms usually remain mild [3]. Visual disturbances may also occur, especially sudden myopia [4], but neurological involvement is scarcely reported. In this case report, we describe and discuss brain involvement of PUUV infection on magnetic resonance imaging (MRI).

## Case Report

A 45-year-old male, with a known history of psoriatic arthritis, was referred to our hospital by his physician, with fever, nausea, headache, asthenia, and visual disturbances. He had competed in a triathlon four days earlier and had been kayaking, cycling, and running in the forest for weeks before.

**Figure 1:**
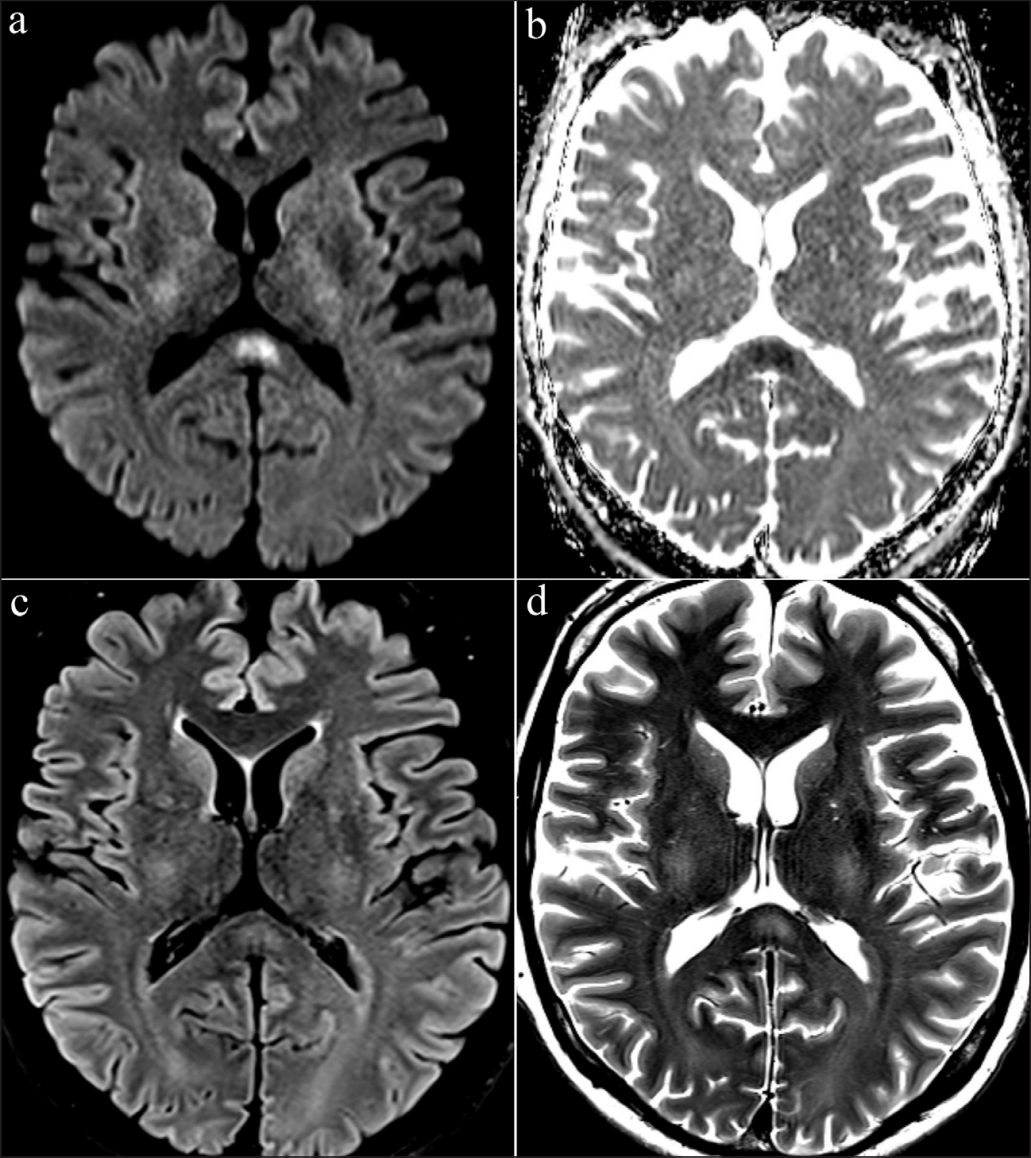
Axial diffusion-weighted image (b = 1000 s/mm^2^) **(a)** showed a high signal intensity in the splenium of corpus callosum. Apparent diffusion coefficent (ADC) map image **(b)** showed a low ADC. Axial FLAIR **(c)** and T2-weighted images **(d)** showed a slight hyperintense signal at the same location.

General clinical and neurological examination were normal. Blood analyses on admission showed increased C-reactive protein (247.9 mg/l) and mild thrombocytopenia (74,000 platelets per microliter), then high creatinine (2.02 mg/dl), high urea (66 mg/dl), and eosinophilia (1600/μl) four days later. Due to increasing headaches, MRI of the brain was performed (Figure 1) to rule out cerebral vein thrombosis. Axial diffusion-weighted image showed a high signal intensity in the splenium of corpus callosum at high b-value (b = 1000 s/mm^2^) with low apparent diffusion coefficient values. Axial T2-weighted and fluid-attenuated inversion-recovery images showed a slight hyperintense signal on the same location. There was no abnormal contrast enhancement, nor cerebral venous thrombosis. Anti-Puumala virus IgM antibodies were detected using enzyme immunoassay, confirming diagnosis of acute PUUV infection [5]. Patient was discharged eight days after admission. The MRI findings had resolved completely in follow-up study three weeks later (not shown). Diagnosis of cytotoxic lesion of the corpus callosum (CLOCC) was made.

## Discussion

Our patient was diagnosed with PUUV infection associated with CLOCC. We found only two papers in the literature reporting similar findings [6,7]. These splenial lesions have been called by many names, including “mild encephalopathy with reversible splenial lesions (MERS)”, “reversible splenial lesion syndrome (RESLES)”, and “reversible splenial lesions”. It has not been without controversy, as encephalopathy is not always mild (can be absent or severe) and also because the lesions are not always completely reversible nor always strictly splenial. On the other hand, it is generally agreed that these callosal lesions with restricted diffusion are caused by cytotoxic edema. Therefore, Starkey et al. recently termed these lesions “cytotoxic lesions of the corpus callosum” (CLOCCs) [8].

CLOCCs have been associated with drug therapy, malignancy, infections, subarachnoid hemorrhage, metabolic abnormalities, trauma, and other entities. The involvement of the corpus callosum typically shows one of three patterns: a small round or oval lesion located in the center of the splenium (our patient), a lesion centered in the splenium but extending through the callosal fibers laterally into the adjacent white matter, or a lesion centered posteriorly but extending into the anterior corpus callosum. Being familiar with the imaging appearance of CLOCCs might help to avoid a misdiagnosis of ischemia [8].

## Conclusion

To the best of our knowledge, this is the third reported case of CLOCC associated with PUUV infection, adding further evidence that PUUV can be associated with CLOCC. Although CLOCCs have been associated with numerous pathologies, PUUV infection should be considered in encephalitis of unclear origin, particularly if sudden myopia and/or renal symptoms occur in a patient at risk of exposure to rodents.
